# Picturing Bravery: A Rapid Review of Needle Procedures Depicted in Children’s Picture Books

**DOI:** 10.3390/children10071097

**Published:** 2023-06-22

**Authors:** Hiba Nauman, Olivia Dobson, Anna Taddio, Kathryn A. Birnie, C. Meghan McMurtry

**Affiliations:** 1 Department of Psychology, University of Guelph, Guelph, ON N1G 2W1, Canada; hnauman@uoguelph.ca (H.N.); odobson@uoguelph.ca (O.D.); 2 Leslie Dan Faculty of Pharmacy, University of Toronto, Toronto, ON M5S 3M2, Canada; anna.taddio@utoronto.ca; 3 Department of Anesthesiology, Perioperative and Pain Medicine, Cumming School of Medicine, University of Calgary, Calgary, AB T2N 1N4, Canada; kathryn.birnie@ucalgary.ca; 4 Department of Pediatrics, Schulich School of Medicine & Dentistry, Western University, London, ON N6A 3K7, Canada; 5 Pediatric Chronic Pain Program, McMaster Children’s Hospital, Hamilton, ON L8N 3Z5, Canada

**Keywords:** children, needle fear, pain, vaccinations, venipuncture, books, bibliotherapy

## Abstract

Existing research has identified evidence-based strategies for mitigating fear and pain during needle procedures; yet, families often experience limited access to health professionals who deliver these interventions. Children may benefit from learning about such strategies in a developmentally appropriate and accessible format such as a picture book. This review aimed to summarize content related to needle procedures represented in picture books for 5- to 8-year-old children. Key terms were searched on Amazon, and the website was used to screen for relevant eligibility criteria. Three levels of screening and exclusions resulted in a final sample of 48 books. Quantitative content analysis was used to apply a coding scheme developed based on relevant Clinical Practice Guidelines and systematic reviews. Cohen’s Kappa indicated strong reliability, and frequencies were calculated to summarize the content. The books were published between 1981 and 2022. All 48 books included at least one evidence-based coping strategy. Distressing aspects such as scary visuals were often included (27.1%), as well as specific expressions of fear (52.1%) and pain (16.7%). Overall, this study paves the way for researchers interested in evaluating the effectiveness of picture books on children’s knowledge and self-efficacy, as well as creating interventions for coping.

## 1. Introduction

### 1.1. Overview

Undergoing needle procedures is a common experience throughout childhood [1]. In fact, children in Canada between the ages of 4 and 6 need vaccinations to start school and then continue to receive 1–2 yearly needles for booster and influenza vaccinations [2]. Since the COVID-19 pandemic, children are also being offered the COVID-19 vaccination and booster doses, which add to the over two dozen needles children may receive by the age of 18 [2]. Unfortunately, over half of children between the ages of 4 and 8 are fearful of needles to some extent with approximately 30% self-reporting high levels of fear [3]. This contributes to negative experiences with needle procedures. Ways to reduce needle fear are needed to prevent such negative experiences and promote vaccination uptake in children. Akin to their use for other health topics, children’s picture books may be an important source of developmentally appropriate education on needles and could be used by parents to help their child cope with their needle fear [4,5,6]. To our knowledge, there is no existing review summarizing the content of children’s picture books with respect to needles.

This study sought to capture what information picture books present to young children (5 to 8 years old) and their caregivers about needle procedures. The primary research question was: What content is available in existing commercially available children’s books regarding needle procedures? This study is part of a larger project aiming to include children’s voices in creating a needle fear intervention that includes a children’s book.

### 1.2. Childhood Needle Procedures and Needle Fear

As part of the recommended vaccination schedule, Canadian children may receive over two dozen needles before turning 18 [2]. According to a recent systematic review and meta-analysis, needle pain and fear are barriers to vaccination in 5 to 13% of children ages 18 and under [7]. Beyond vaccinations, children may also be fearful of other necessary needle procedures such as blood draws, which are used to monitor their health [8].

Needle fear is best understood as existing on a spectrum ranging from mild fear, which is normative, to high and clinically significant levels of fear [1]. While clinically significant levels of needle fear may warrant a diagnosis of Blood-Injection-Injury Phobia [9], high but subclinical levels of needle fear are also concerning due to their associated consequences. For example, highly fearful children may cry, freeze, or attempt to escape needle procedures [1]. This may impede the safe completion of the procedure. 

High levels of needle fear are also associated with longer procedure times, increased pain, distressing memories related to the procedure that predict perceived pain at future appointments, and even ineffective pain management [1,3,10,11]. This creates a problematic cycle where fear and pain exacerbate each other and lead to adverse experiences with significant longitudinal implications. In fact, children with needle phobia may even need to be sedated to undergo routine needle procedures, which can entail additional risks [12]. Highly fearful children may also grow up and become parents who model needle fear to their own children, which further perpetuates this cycle [1]. As such, it is important to understand what resources exist that adequately equip young children and their caregivers with strategies to manage needle fear in order to “break the cycle” and ensure that needles that are important for both individual health (i.e., diagnosis, monitoring, treatment) and community health (i.e., community immunity from vaccination) are not delayed or avoided. The latter is critical following the COVID-19 pandemic, which highlighted the urgent need for targeted interventions that address needle fear as a barrier to vaccinations [7].

### 1.3. Needle Fear and Pain Management Strategies

Systematic reviews and resulting Clinical Practice Guidelines have identified evidence-based strategies for mitigating fear and pain during needle procedures for children. These strategies vary based on the level of fear (i.e., low to moderate vs. high), procedure type (e.g., vaccination), and type of intervention (e.g., psychological). Specifically, guidelines and systematic reviews exist for:

(1) Low to moderate fear and needle pain management for vaccinations [13,14];
(2) Exposure-based treatment for individuals with high levels of needle fear in the context of vaccinations [15,16];
(3) Psychological interventions for needle procedures generally [17].

As venipuncture-specific guidance is more limited, recommendations for addressing children’s fear and pain during venipunctures can be extrapolated as relevant from guidance for vaccinations [13,14,15,16] and needle-related procedures generally [17,18].

Best practices for managing needle pain and fear from this evidence base are summarized in Table 1 according to the level of fear (high fear vs. low to moderate fear). For high levels of needle fear, exposure-based therapy (EBT) is recommended prior to the use of more typical pain and fear management strategies; however, for young children, the evidence is limited [15]. Muscle tension is another approach targeted towards those with high levels of needle fear who also experience vasovagal reactions such as dizziness and fainting [15]. EBT is considered the gold standard treatment for specific phobias [19,20] and involves gradual exposures in which patients face their feared stimuli or situation from least (e.g., seeing an illustrated needle in a book) to most feared (e.g., undergoing a vaccination) [21]. Receiving EBT from a trained professional is inaccessible for many due to the associated financial costs, lengthy waitlists, a shortage of trained clinicians, and social stigma [22]. Consequently, there is a serious need for more widely accessible interventions to support children with needle fear with learning how to manage their fear and cope during the procedure.

Low–moderate fear and pain management strategies for children ages 5 to 8 years old are grouped within the “5P” approach. The “5Ps” include process (e.g., clinician and parent education), procedural (e.g., avoiding aspiration for vaccinations), physical (e.g., positioning), pharmacological (e.g., topical anesthetics), and psychological interventions (e.g., distraction) [14].

### 1.4. Bibliotherapy and Children’s Picture Books

A promising method to support children with managing needle pain and fear is to equip them with information and coping strategies in a developmentally appropriate and engaging format that is conducive to their learning. More specifically, child and parent education about the other strategies presented in Table 1 could be achieved through children’s books. Books are a tool that can promote insight into personal challenges [23], and this has been known in the literature as “bibliotherapy”. In fact, books have been considered helpful for describing medical information to children as they provide both written and visual information concurrently, which may help enhance children’s comprehension, memory, and retention [24]. More specifically, children’s books have been used to provide developmentally appropriate education on anxiety [22,25] and health difficulties ranging from asthma to physical injuries and developmental disabilities [4,5,6]. They have also been used to facilitate children’s understanding of complex topics such as cancer, death, and grief [26,27,28], as well as components of medical procedures such anesthesia [29].

Tsao and colleagues created a picture book for pre-school-aged children in Taiwan that depicted a character undergoing a venipuncture [30]. They found that children in the picture book intervention group had significantly lower behavioral distress before, during, and after the venipuncture than children in the control group. While there may presently be other picture books that intend to prepare children for needle procedures [30], no known studies have summarized the included content and how consistent it is with evidence-based guidelines. It is important to explore what is currently available in children’s picture books that depict needle procedures to understand what is available for caregivers to support their children with managing their needle fear. Identifying strengths and relevant gaps in the books may ultimately inform the development of an evidence-based resource (e.g., a children’s picture book) that bridges knowledge to action gaps related to managing children’s pain and fear during needle procedures [31,32].

### 1.5. Objectives

The overarching objective of this study was to explore how existing children’s picture books present needle-related information and experiences using a rapid review methodology. Rapid reviews are an emergent method used to collect, analyze, and interpret evidence by streamlining or omitting certain steps to produce evidence in a resource-efficient, but systematic manner [33,34,35]. This methodology allowed us more flexibility to screen for and capture children’s picture books in comparison to other methods such as systematic or scoping reviews. By taking a rapid “snapshot” of children’s picture books on a platform that is constantly changing and updated (i.e., Amazon), this review could more quickly synthesize the evidence and inform interventions for needle fear.

The specific objectives of the review were to describe the content of children’s books related to:

(1) What to expect during needle procedures (e.g., type of procedure, setting, people present);
(2) Educational information related to needles (i.e., importance of needles, procedural and sensory information, adult-directed material for caregivers);
(3) Expressions of needle fear and pain (i.e., crying, requesting support, verbal expressions (e.g., “that hurt!”, “I don’t want it!”));
(4) Strategies for coping during needle procedures (i.e., evidence-based);
(5) Unhelpful strategies and fear-inducing depictions of needles (i.e., not evidence-based strategies).

Given the exploratory nature of this study, no specific hypotheses were made.

## 2. Materials and Methods

### 2.1. Eligibility Criteria

This review is part of a larger line of research on the creation of a needle fear intervention through a children’s book. Eligible books had to be picture books available for purchase in North America with a target age within 5 to 8 years. While picture books are also used by children ages four and under, this age range was excluded due to their more limited ability to report on future internal states different from their current state (e.g., anticipated fear) [36], which is an important component of the larger line of research.

Beyond the target age, to be eligible, the books had to reference in text and/or visually depict a vaccination or venipuncture procedure (e.g., blood draw). Needles associated with chronic conditions (e.g., finger pricks and insulin injections for diabetes, bone marrow aspirations and lumbar punctures for cancer, etc.) and needles provided at the dentists or vet were excluded as they would have differed in terms of children’s experiences, information needs, and the roles of parents and healthcare providers. Due to the geographical and cultural parameters of this study, only books in English were included. The specific screening criteria categories can be seen in Appendix A.

### 2.2. Search and Screening

**Figure 1:**
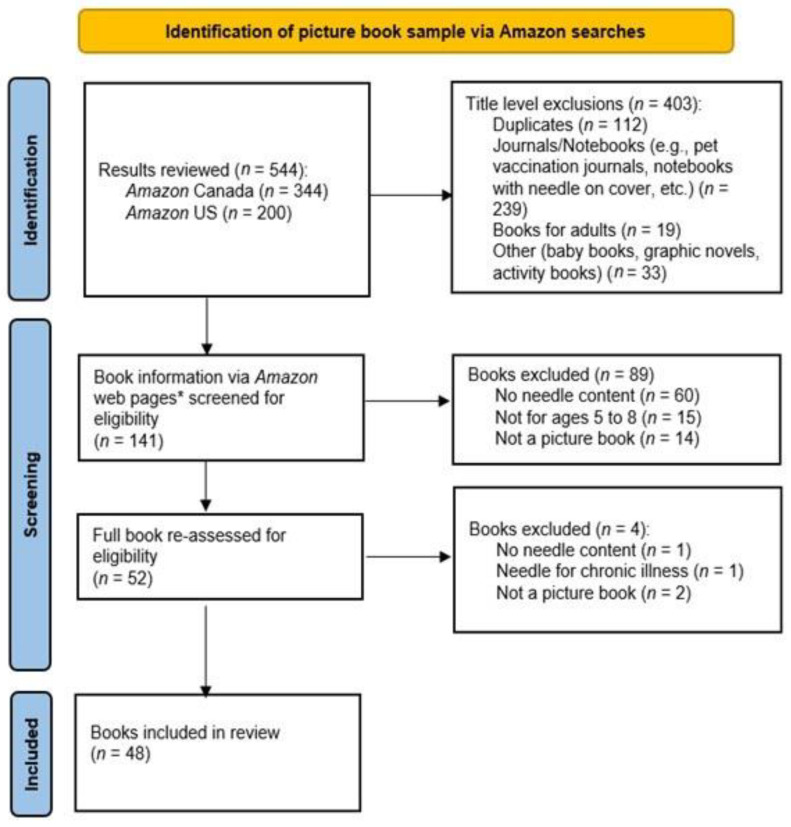
Prisma flow diagram of book sample selection and screening method. * Amazon web pages include information such as author(s), cost, and options for ordering. They also include additional information about the book including, but not limited to book description, age range, reading level, reviews from purchasers, and book previews.

Two primary key terms (“Needle and Health”, “Doctor and Shot”) and three secondary key terms (““Needle” and “Vaccine””, “Vaccine”, “Check up”) were searched on Amazon, an online retailer that has been used to identify a sample in other similar studies [37,38,39]. The searches were conducted between 19 May and 23 May 2022. The first 100 results were reviewed for primary key terms followed by the first 50 results from secondary key term searches. Key terms were searched in the order listed above within the Children’s book section on Amazon Canada (primary, then secondary) followed by Amazon US (primary only due to repetitions). The picture book and English language filters were also turned on to refine each search. Search results were reviewed in the order featured on the website with any sponsored results automatically excluded; of note, the sponsored exclusions were not tracked; however, books that were “sponsored” (and, therefore, excluded) could also show up within the search results if relevant, and these were considered for inclusion. The books were first screened via Amazon web pages; for books meeting the inclusion criteria, the full book was obtained by purchasing a hard copy or e-book through Amazon or from a local library, when available. Full books were then assessed according to the previous screening criteria. Figure 1 outlines each stage of the screening process.

### 2.3. Analyses

Quantitative content analysis was used to evaluate the final sample of included picture books. This method is a systematic approach to describing the presence of specific content in the data [40,41,42]. This approach is typically used to analyze questionnaires or interviews, whereas this study examined children’s picture books. As such, our analyses involved assessing visual depictions of needle procedures, as well as important aspects of the narratives surrounding the procedure. We coded for both visual depictions and narrative/textual descriptions and did not distinguish between them, unless otherwise noted.

Researcher-developed deductive coding schemes for the picture books (Appendix B) and adult-directed supplementary material (Appendix C) were developed for the analysis based on needle pain and fear management Clinical Practice Guidelines and systematic reviews [14,15,16,17], the Child-Adult Medical Procedure Interaction Scale–Revised [43], an unpublished honors thesis on the depiction of needles in picture books, and expert input from study authors. The CAMPIS-R [43] is an observational behavior rating scale commonly used to capture adult and child behaviors during procedural pain, including child distress and coping, as well as adult “coping promoting” behaviors (e.g., distraction, command to engage in a coping strategy) and adult “distress promoting” behaviors (e.g., reassurance, criticism, apology). Thus, the CAMPIS-R informed categories related to depictions of commonly used, but unhelpful (not evidence-based) adult behaviors, as well as depictions of needle recipients that may impede coping (e.g., crying, verbal resistance).

For coding the children’s books, the primary investigator (H.N.) and two research assistants completed a training on the reference materials, coding scheme, and quantitative content analysis. All three individuals initially coded approximately 15% of the sample (i.e., 7 books). Coding was dichotomous for most categories (i.e., presence or absence of category) with some exceptions. For example, ominous or scary visual depictions of needles were coded on a 0–10 numerical rating scale (“no scary depictions” = 0, “mild” = 1–3, “moderate” = 4–6, “high” = 7–10). Cohen’s Kappa and percent agreement were used to calculate intercoder reliability (ICR) for each category. Percent reliability was calculated in addition to Cohen’s Kappa due to the latter being overly stringent for dichotomous coding (i.e., rating responses as either “0” or “1”). The lower end of the range in the Kappa values can be explained by there being a discrepancy in coding between raters on a category with very few responses. 

Deriving ICR for each specific category allowed the primary investigator to assess and identify categories that needed refinement (e.g., categories < 0.7 were revisited); necessary modifications were made to the coding scheme. Refinement of categories included thorough discussion and consultation with the primary investigator’s supervisor (C.M.M.). Following the changes, the three coders re-coded the initial 7 books, as well as 3 more (i.e., 21% of the sample). Kappa and percent agreement were calculated again, and another round of minor revisions was made to the coding scheme through the same process. The two coders with the highest reliability then coded the remainder of the data. The final Kappa values from these two coders on the entire sample indicated strong overall reliability (*M* = 0.78; Median = 0.79; range: 0.43 to 1.0) and so did the percent agreement (*M* = 94.8%; Median = 95.8%; range: 77.1% to 100%).

A consensus-based coding approach was used to code the adult-directed supplementary material. Two authors of this work (A.T. and C.M.M.) who are also lead authors on the CPGs and systematic reviews on managing needle fear and pain, coded the adult supplementary sections together. Any disagreements were discussed until resolved. Frequencies were then calculated to summarize aspects of how needle procedures and related information are depicted in the sample of books.

## 3. Results

### 3.1. Overview

The sample included 48 children’s picture books (see Table 2 for the titles) with content related to needle procedures (see Figure 1). Unless otherwise noted, all analyses included the full sample of books (i.e., *n* = 48).

**Figure 2:**
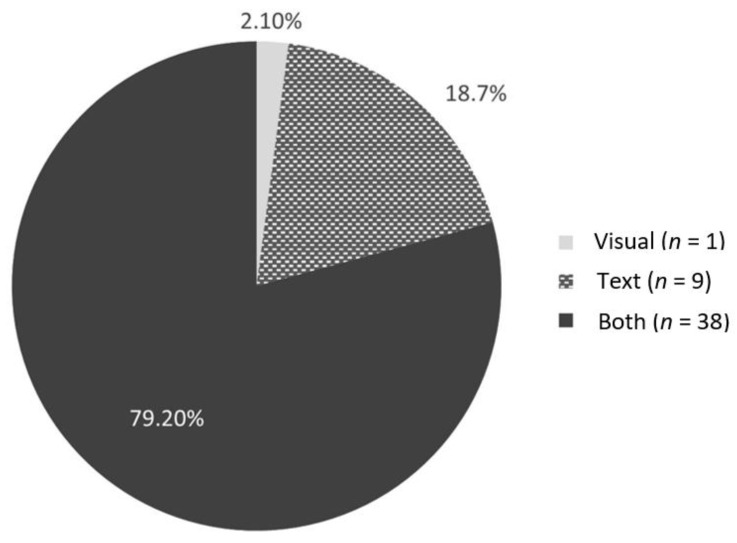
Proportion of needle procedures depicted visually through pictures only, described in text only, or referenced both visually and within text (*n* = 48).

The books were published from 1981 to 2022; however, most (81.3%, *n* = 39) were published after 2015. The majority (64.6%; *n* = 31) are available in both hard copy and e-book formats. The average cost of the hard copy books was CAD 14.11 (Median = CAD 12.99, range = CAD 6.92 to 25.77), whereas the average cost of the e-books through Kindle^TM^ was CAD 4.15 (Median = CAD 6.19, range = CAD 0.99 to 9.99). Needle-related information and experiences were most often represented both visually and in text (Figure 2), and the majority of the sample (77.1%, *n* = 37) featured a character and narrative. A small number of books (6.3%, *n* = 8) did not depict a story line and were primarily educational information for children.

### 3.2. Objective 1: What to Expect during Needles

**Figure 3:**
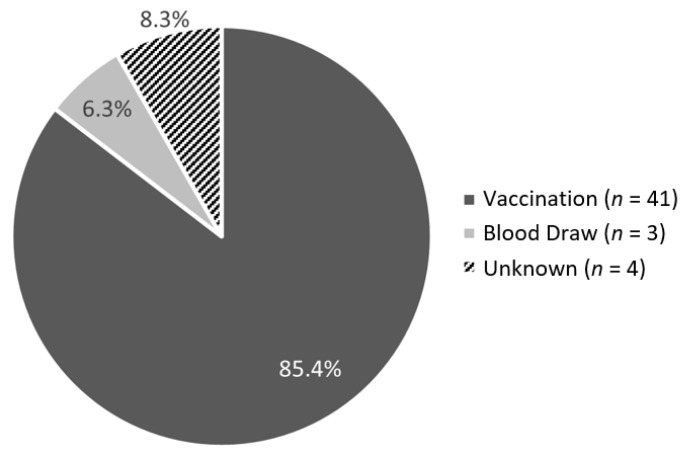
Types of needle procedures represented within the sample (*N*_total_ = 48).

Figure 3 represents the breakdown of the types of needle procedures referenced in the sample, with the majority being vaccinations (85.4%, *n* = 41). The setting of each book was also examined to understand the context within which needles are typically presented. Most needle procedures (75%, *n* = 36) were set in a doctor’s clinic, hospital, or general exam room setting. Three books (6.3%) depicted the needle procedure in a school setting. No needle procedures were set within the home. The remaining books (10.4%, *n* = 5) fell into the “other” category and depicted random or unclear locations (e.g., field, boat, dream, etc.). In 66.7% of the sample (*n* = 32), only the needle procedure was referenced or depicted, whereas almost a third of the books (31.3%, *n* = 15) also included other procedures that commonly occur at doctor’s appointments such as checking height and weight, vision, breathing, and testing reflexes.

**Figure 4:**
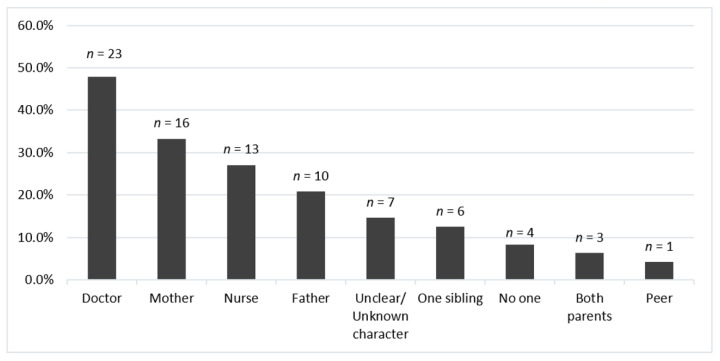
Frequencies of characters other than the child present during needle procedures (*N*_total_ = 48). The following character categories had frequencies of 0%: multiple siblings, teacher, grandparent(s), child life specialist, mental health provider, pharmacist.

Regarding characters present during the procedure, a caregiver figure was present in 45.8% of the books (*n* = 22); of these, mothers were represented the most (72.7%, *n* = 16). Within clearly depicted healthcare providers, only doctors and nurses were represented. The list of coded characters is reflected in Figure 4.

### 3.3. Objective 2: Educational Information about Needles

In regard to what children are learning about needles, sensory (i.e., what it will feel like) and procedural (i.e., what will happen) information was provided in 60.4% (*n* = 29) and 39.6% (*n* = 19) of the books, respectively. The importance or rationale for receiving a needle was explained in 81.3% (*n* = 39) of the books. Education about the procedure that was given in advance of the needle (e.g., at home, in the car, waiting room, etc.) was provided to the character or reader in approximately half the sample (52.1% *n* = 25).

Beyond the children’s story, 16.7% of the books (*n* = 8) featured an additional educational section targeted towards adults, which are summarized in Table 2. These adult-oriented sections ranged from 1 page of bullet point tips for getting a needle to a near 30-page guide outlining strategies to support caregivers of children with varying degrees of needle fear. The expertise of book authors who included supplementary adult-targeted material was also reviewed, and 62.5% of the books had an author (or authors) with relevant credentials or experience (e.g., child-life specialist, pediatrician, child psychologist, play therapist, etc.). Within the eight books, 50.0% (*n* = 4) provided a definition, description, or explanation of the fear of needle procedures that appeared to be accurate. Four books (50%) provided an explanation of how vaccinations work, and only 25.0% (*n* = 2) explained community (herd) immunity in a manner that seemed accurate. Fifty percent (*n* = 4) of the books with adult material referenced the use of mental visualization or imagination to cope with needle fear, and two books (25.0%) referred to providing children with a desirable item or experience following successful completion of a needle procedure (i.e., reward). Using cold vibration and topical anesthetics was also referenced in 25.0% (*n* = 2) of the adult sections. In the majority of adult-oriented sections (87.5%, *n* = 7), targeted parent and child education were included in many forms including lists with tips and guidance on how to use strategies (e.g., blowing bubbles, visualization). 

Other helpful strategies referenced included parent presence during the needle (62.6%, *n* = 5), using neutral wording to signal the procedure (12.5%, *n* = 1), and avoiding excessive reassurance and false suggestions (50.0%, *n* = 4). Distraction was a key strategy present generally in 37.5% (*n* = 3) of the adult guides. Distraction using breathing specifically was included in 25.0% (*n* = 2) of the adult sections. Exposure-based therapy/modelling was referenced in only one of the adult sections (12.5%). None of the supplemental adult sections included references to evidence-based strategies such as avoiding aspiration, avoidance of restraint, providing the most-painful needle last, and using upright positioning, muscle tension, or applied tension. Qualitative impressions of each adult supplemental section are included in Table 2.

### 3.4. Objective 3: Expressions of Fear and Pain 

In examining how specific expressions of fear and pain were represented within our sample, we found that approximately half of the full sample (52.1%, *n* = 25) included characters verbally expressing their fear of the needle procedure (e.g., “I’m scared”, “I’m afraid of needles”, etc.). In contrast, pain was verbally expressed in 16.7% of the books *(n* = 8). Verbal expressions of reassurance and/or false suggestion (e.g., “don’t worry, it won’t hurt”, “it will be fine”) were included in 29.2% of the books (*n* = 14). In 29.2% of the sample, the needle recipient sought information about the needle (e.g., “why do I need it?”, “will it hurt?”, “when will it end?”) (*n* = 14). Depictions and/or descriptions of crying, and verbal resistance (e.g., “I don’t want it”, “stop!”) were each included in 20.8% of the books *(n* = 10). Only one (2.1%) book depicted and/or described a character requesting emotional or physical support (e.g., “can you hold my hand?”, “hold me!”). In contrast, 12.5% (*n* = 6) of the sample depicted and/or described distress in some other manner (e.g., running away during procedure, hiding from needle, etc.).

### 3.5. Objective 4: Coping Information for Needle Procedures

**Figure 5:**
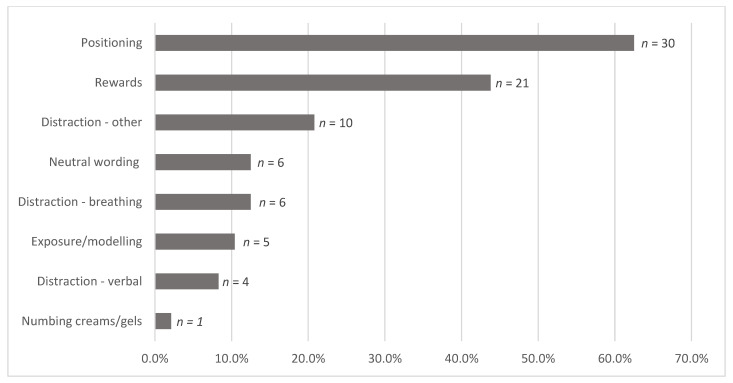
Evidence-based and other helpful coping strategies (*N*_total_ = 48). The following strategy categories had frequencies of 0%: muscle tension, distraction: media.

All 48 books (100%) included at least one evidence-based strategy for coping with needle fear, as reflected in Figure 5. Using upright positioning was commonly depicted (>60%). Forms of distraction beyond breathing, media, and verbal distraction were depicted or described in a fifth of the sample (20.8%, *n* = 10) and most often included guided imagery or visualization (e.g., picture self as a brave lion, imagine fish swimming in ocean), or singing. No distraction via media or technology (e.g., tablets, VR, phones, etc.) was depicted.

### 3.6. Objective 5: Unhelpful Strategies and Fear-Inducing Depictions

**Figure 6:**
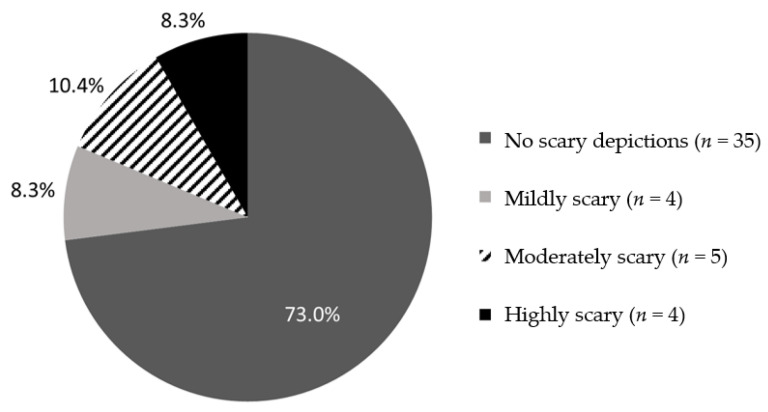
Frequency and intensity of scary visual depictions in children’s books as coded from “no scary depictions” = 0, “mild” = 1–3, “moderate” = 4–6, to “high” = 7–10 (*N*_total_ = 48).

Strategies that are not promoted in Clinical Practice Guidelines and systematic reviews included the use of ice, vapocoolants, and oral pain medication. The use of ice was depicted in one book (2.1%), whereas the other aforementioned strategies were not referenced. Just over one quarter (27.1%; *n* = 13) included ominous or scary visual depictions (e.g., unrealistically large needles, dark or scary visuals, etc.); see Figure 6. In contrast, the presence of exaggerated or scary *written* descriptions were found in 8.3% of the books (coded dichotomously). Specific statements included that are associated with escalating distress included: apologies (2 books; 4.2%), simplified reassuring statements and false suggestions (e.g., “don’t worry”, “it won’t hurt”), which came up in 29.2% (*n* = 14) of the books, as well as criticism (“why are you crying so much?”), which was not present in any book. The use of restraint was not depicted in any of the 48 books.

## 4. Discussion

### 4.1. General Discussion

To our knowledge, this review is the first to explore content related to needle procedures in children’s picture books. The sample of picture books provided a “snapshot” of the kind of information children may receive at an early age in relation to needle procedures. Overall, vaccinations made up the majority of needle procedures included. This is unsurprising as most Canadian children ages 5 to 8 in Canada require mandatory vaccinations to attend school [2], making it a more pervasive procedure than childhood venipunctures. Yet, needle fears that stem from vaccinations may go on to generalize to other stimuli or procedures such as venipunctures [8], which were largely unrepresented within the books. Most needle procedures were also set in clinic, hospital, or general exam rooms, which is consistent with where needle appointments often take place. Mass immunization settings, which have grown in prevalence over the last few years [92], were not represented in the sample despite some books being published during the COVID-19 pandemic. A strength observed in most books (81%) was including an explanation for why needles are important. Many of these explanations were specific to vaccines, how they work, and what their function is on an individual and community level. Being exposed to the purpose and function of vaccinations may contribute to building children’s trust in their effectiveness. This is important because a driving factor of vaccine hesitancy is confidence and trust in the safety and effectiveness of vaccinations [93]. While having information about and an understanding of vaccinations early on may help protect children from the readily accessible negative concerns and unverified medical information that exists on online platforms [94], such rationale-based information is insufficient on its own. Children must be provided with information on how to prepare for and cope with the needle procedure. For example, 16.7% of the books targeted caregivers directly by providing them with educational information or tips at the end of the book, some of which they could directly use to teach their child coping strategies. This supplementary information varied in length (e.g., 1 page of tips, 30-page parent guide) and quality (vague information, contradictions, confusing). This is disappointing as caregivers are in the unique position to help prepare their child for the procedure beforehand, as well as support in coping during the procedure. It is evident that caregivers may benefit from a practical, evidence-based, structured guide for supporting their child with needle pain and fear.

Consistent with recommendations for vaccinations [13], many books provided information about how the needle procedure might feel (sensory information) and what might happen (procedural information). Interestingly, the former information was provided more often (60.4%) than the latter (39.6%). According to a study exploring 7- to 10-year-old children’s perspectives on physician check up’s/wellness visits, children who endorsed higher ratings of fear were more likely to want increased preparatory information and participation [95]. As such, authors seeking to prepare children for needles through books should consider including more descriptive and step-by-step information about the procedures (e.g., describe tying a tourniquet, cleaning insertion site, etc.).

Given the focus of the pediatric literature on pain during needles, it was surprising that verbal expressions of pain were only reported in 16.7% of the books. A potential explanation may be that authors are hesitant to make direct references to the inherently painful nature of pain due to concerns around instilling fear in children. In exploring what children may learn about coping with needle pain and fear from our sample, we noticed that several depicted strategies were consistent with existing systematic reviews and Clinical Practice Guidelines [13,14,15,16,17] including: upright positioning of the character receiving the needle, use of neutral wording, modelling and/or exposure (refers to modelling or exposure (“facing fears”) in advance of the needle procedure (e.g., showing character a needle before procedure, watching a video of needle before the procedure, demonstrating giving the needle to a stuffed toy, etc.)), the use of topical anesthetic creams or gels, and distraction using breathing. It was surprising that the books did not depict distraction techniques that involved media or technology of any kind (e.g., tablet, phone, virtual reality, etc.) given the accessibility of such technology and increasing empirical evidence [96,97].

In conjunction with evidence-based strategies, this study also aimed to examine depictions and descriptions of strategies that may be unhelpful or promote distress in children. We observed that many books had a “check up” narrative and depicted other procedures (e.g., reflex test, vision test) in addition to a needle procedure. This could be helpful in that needles are placed in a broader context and procedural information is being provided about these aspects. However, such content may also be difficult for fearful children to consume as a subset of children fear many aspects of their check up appointments beyond needles such as ear checks, throat swabs, and the use of the tongue depressor [95]. Examining these procedures and how children may perceive them was beyond the scope of this review; this should be considered in future research.

While evidence-based recommendations advise against making false suggestions to children such as “it won’t hurt”, 29.2% of the books included such reassuring statements and false suggestions. While research has emphasized the importance of being honest and preparing children for the sensory components of the procedure [1,36], it is unclear how directly children should be informed and educated about needle pain. Future research should explore how children perceive educational information about needle pain and its impact on their fear of needle procedures.

### 4.2. Strengths, Limitations, and Future Research Directions

This review has a number of strengths including the use of a rigorous coding scheme developed from existing systematic reviews and Clinical Practice Guidelines [13,14,15,16,17], overall good reliability between coders indicated by Kappa values and percent agreement, and consensus-based coding of adult material by two of the lead authors of the aforementioned reviews and guidelines. Considerations of access were also at the forefront of this work. The Amazon website, which was carefully selected to identify the book sample, is more broadly accessible to parents in North America than traditional approaches (e.g., library catalogues, which may vary by community and region). More than half the sample was accessible in both hard copy and e-book format, with the latter being more affordable for families. This review also identified multiple books that caregivers can use to prepare their children for their needle appointments. Educating children in this manner may serve as a promising approach for reducing children’s distress during needle procedures, as demonstrated by Tsao and colleagues [30].

This review also has some limitations. While the sample was identified in May 2022, searches related to accessibility (i.e., book cost and format) were conducted in January 2023. There may be newer books or other evidence-based books that were not available through Amazon and, thus, could not be included. While some e-books may be accessed for “free” with an Amazon Kindle subscription, the sample did not include free books that would be accessible to low SES families and those who do not have an Amazon subscription, account, or access to the Internet. Another limitation concerns the focus of the coding scheme: the picture books were reviewed for very specific visual content and types of written statements, as opposed to a full thematic analysis of each book. While the latter would more fully identify the themes or “lessons” children are taking away from the books (e.g.: fears can be overcome; needles are here to help), capturing such abstract and varying narratives was beyond the scope of this review because: (a) they cannot be coded in a systematic manner consistent with the methodology required of a rapid review, and (b) we cannot infer the themes that would be most salient to children. Likewise, while this review considered how “scary” and “ominous” the books were, despite good reliability in the coding, variability was observed in what each coder found to be “scary” because fear is subjective. Thus, in future research, children should be asked to directly review and report on relevant themes and the depiction of needles in picture books as adults’ perspectives on what they think children learn or fear may be skewed and inaccurate. Furthermore, given that we did not code for socioeconomic class, religion, language (all books were in English), or ethnicity, understanding representation in children’s books related to needles remains an important area for future research.

While educational videos, educational pamphlets, and a multifaceted CARD™ System have been used to make evidence-based strategies for coping with needle fear and pain actionable [98,99], the CARD™ System is the only known widespread intervention that has been evaluated and to have demonstrated effectiveness and utility [100,101]. Without a systematic evaluation of the picture books, we cannot infer that any of them are effective in supporting children with their needle fear and pain, despite depicting evidence-based strategies. 

Additionally, the study only screened for picture books written in English and available in North America. While examining books in other languages was beyond the scope of the study, it is important to recognize that other picture books describing and depicting needle procedures likely exist in other languages. Although differences in the provision of needles between regions and cultures may be challenging to represent in a single picture book, children globally would likely benefit from a free and accessible book that is consistent with evidence-based recommendations and can be translated easily.

Overall, this review will help researchers understand how needle procedures are presented to young children currently and what gaps may need to be addressed in an intervention that uses bibliography to support children with needle fear and their caregivers. For example, given the absence of depictions of mass vaccination clinics, authors should consider whether it may be beneficial to represent this unique vaccination setting in future books. This may be particularly important because mass immunization clinics are often overwhelming for highly fearful children, as well as conducive to experiencing Immunization Stress-Related Responses [102,103]. More generally, interventions that promote self-efficacy in caregivers and children may help reduce reliance on mental health professionals to treat needle fear and help prevent a myriad of consequences such as healthcare avoidance, modelling fear to others, and ineffective pain management [1,3]. Further research is needed to determine whether this form of educational intervention will appropriately meet the needs of children requiring support and whether this type of content can lead to improved management of pediatric needle fear and pain.

## 5. Conclusions

The present study is the only known review of picture books for 5- to 8-year-old children that depict or describe needle procedures. The results provided a “snapshot” of the kind of evidence-based information related to needle procedures currently accessible to young children and their caregivers. Overall, this study serves as an important first step towards the development of an e-resource for caregivers and children to gain the information and strategies needed to manage needle fear and pain more independently at home.

## Figures and Tables

**Table 1:** Recommended needle fear and pain management strategies for children ages 5–8 with other age specific information noted as relevant.

Strategy	Timing	Recommendation	Key Citations
Strategies for High Levels of Needle Fear with or without a Vasovagal (Fainting) Response			
Exposure-Based Therapy (EBT)	Well before day of the needle	For children ages 7 and above with high levels of needle fear, EBT is recommended. In fact, EBT is considered the gold standard treatment for specific phobias such as blood-injury-injection phobia.	[13,15]
Muscle Tension during Exposures	Well before the day of and during the needle	For children ages 7 and above with high levels of needle fear and a history of fainting, practicing muscle tension during exposures is recommended.	[13,15]
Strategies for Low to Moderate Fear and Pain			
Clinician Education	Before the day of the needle.	It is recommended that clinicians administering the needle be educated on implementing needle pain and fear management strategies.	[13]
Parent Education	Before and on the day of the needle.	Educating parents on how to mitigate their child’s pain and fear both before and on the day of needle is recommended to increase the use of interventions during the needle.	[13]
Child Education	Before and on the day of the needle.	Children ages 3 and up should be provided procedural (e.g., what will happen?) and sensory (e.g., how will it feel?) information and be taught how to use coping strategies. Such information should mostly be provided in advance. On the day of the procedure, children should be provided with neutral information and coping strategies.	[13]
Avoid Reassurance and False Suggestion	Before and during the needle.	Repeated and simplified reassurance and false suggestions (e.g., “don’t worry”, “it’s okay”, “it won’t hurt”) can increase children’s distress and pain.	[13]
Using Topical Anesthetics	20 to 60 min before the needle.	Topical anesthetics block the transmission of pain signals from the skin (McLure and Rubin, 2005) and are recommended. They can be in the form of numbing creams, gels, or patches.	[13]
Muscle Tension	Immediately before, during, and after the needle.	Muscle tension is recommended for children ages seven and above with a history of fainting.	[13,16]
Combination of Cold and Vibration (e.g., Buzzy Device)	Immediately before and during the needle.	The Buzzy Device, which applies vibration and cold above the needle insertion site, has been shown to be a promising intervention for reducing procedural pain and anxiety.	[18]
Neutral Wording to Signal Procedure	Immediately before the needle.	It is recommended to use neutral wording to signal the impending procedure to children (e.g., “here we go!”).	[13]
Distraction	Before and during the needle.	It is recommended that distraction be used to draw children’s attention away from the needle. For children ages 3 to 12, verbal, video, and music distractions are recommended.	[13]
			[17]
Positioning: Sitting Upright	During the needle.	It is recommended that children ages 3 and older sit upright, rather than be lying in a supine position. This positioning has been shown to decrease fear and observed distress in children.	[13,14]
Avoid Aspiration (Vaccinations Only)	During the needle.	Aspiration should *not* be used during intramuscular vaccine injections as the technique can increase pain.	[13]
Parent Presence	During the needle.	Parents of children aged 10 and younger should be present during the child’s needle.	[13]
Breathing with Distraction	During the needle.	Breathing interventions, such as coughing, are recommended against. Instead, it is suggested that children breathe with a toy distraction (e.g., pinwheel, bubbles, etc.).	[13]
			[17]
Most-Painful Needle Last	During the needle.	As some vaccines are more painful than others, and many children receive multiple injections at a single visit, the most-painful needle should be administered last.	[13]

**Table 2:** Summary of picture book sample, book costs, strategies included, and overall comments.

Book Title	Citation	Price: Hardcopy/Kindle ($CAD)	Evidence- Based Strategies	Distressing Aspects	Adult Material	Comments
*Ahana Got a Vaccine*	[44]	12.99/NA	✔ ^1,5,6,7,8,9,10^	✔ ^b^	--	Includes multiple evidence-based strategies.
*Alpha Vaccination; an Animal Alphabet Vaccination Story*	[45]	9.99/2.99	✔ ^1,6,7,8,9^	✔ ^b,d^	--	Provides some helpful coping information. Many scary depictions of needles sticking out of animals.
*Betty’s Blood Test*	[46]	13.08/3.87	✔ ^1,3,6,7,8,9,10^	✔ ^b^	--	Represents a blood draw and provides helpful strategies and character education.
*Bobby the Bulldog Goes for a Blood Test*	[47]	10.24/NA	✔ ^2,6,7,8,9,10,11^	✔ ^b^	--	Represents a blood draw and includes the use of modelling and numbing cream.
*Boomer Get His Bounce Back*	[48]	6.82/4.99	✔ ^9,10^	--	--	Includes a character running and hiding from a needle until provided with support and education.
*Brave Bora*	[49]	9.00/NA	✔ ^1,3,6,9,11^	--	--	Includes multiple evidence-based strategies.
*Brave Jake: A Story about Being BRAVE at the Doctor*	[50]	13.03/7.25	✔ ^1,4,5^	--	--	Ambiguous needle procedure presented. Includes distraction using breathing. Developmentally appropriate visuals.
*Brave Kayla:* *A Story about Being BRAVE at the Doctor*	[51]	10.29/8.02	✔ ^1,4,5^	--	--	Ambiguous needle procedure presented. Includes distraction using breathing. Developmentally appropriate visuals.
*Did You Know Shots Give You Superpowers*	[52]	11.05	✔ ^1,7,8,9^	✔ ^b^	--	Includes a detailed explanation of how vaccinations work and developmentally appropriate visuals.
*Don’t Be Afraid*	[53]	18.71/5.44	✔ ^1,6,7,8,10^	✔ ^b^	--	Features many helpful strategies such as distraction, caregiver presence, and rewards.
*Dr. Ridicudopickudopot and the Shot*	[54]	13.59	✔ ^6,9^	✔ ^b,c,d,e^	--	Features an immensely clumsy and untrustworthy doctor, which contributes to highly scary visuals.
*Ellie is Vaccinated*	[55]	16.23/7.69	✔ ^1,6,7,8,9,10,11^	--	--	Several evidence-based strategies represented and developmentally appropriate visuals.
*Facing Your Fear of Shots*	[56]	9.29/7.44	✔ ^1,9,10^	--	--	Includes real pictures, which may help set up realistic expectations about the procedure. Includes also a child-friendly glossary of terms.
*Fish are Not Afraid of Doctors*	[57]	6.99/6.99	✔ ^1,6,8,9^	--	✔ “Note to Caregivers” section includes some accurate, but vague information. Educates parents on visualization, relaxation, and distraction. Certain wording (“no one likes getting shots...”) may promote negative attitudes towards needle procedures.	Character uses visualization and their imagination to cope with their fear. Developmentally appropriate visuals are included.
*Froggy Goes to the Doctor*	[58]	10.88/7.99	✔ ^6^	✔ ^c,d^	--	Represents an untrustworthy doctor holding a giant needle and making a joke to scare the needle recipient. Such scary visuals may potentially promote fear in children.
*Gracie Gets A Shot*	[59]	12.57/9.06	✔ ^1,5,6,8,10^	✔ ^b,c,d^	--	Initially describes and depicts needle pain in a scary way (e.g., “a thousand bee stings”). Includes some helpful strategies (e.g., counting down procedure).
*How Do Shots Work*	[60]	4.0/2.99	✔ ^1,4,7,8,9,10^	✔ ^b^	--	Represents several evidence-based strategies and includes a detailed explanation of how vaccinations are important for fighting viruses.
*It’s Time For Your Checkup: What to Expect when going to a Doctor Visit*	[61]	14.34/3.32	✔ ^1,4,6,7,8,9,10^	--	✔ Includes a “Note to Parents” section containing coping strategies for different age groups. Options for distraction are also provided by age range. The tips provided are concrete and consistent with Clinical Practice Guidelines. Includes also a discussion on looking at or away from the needle and examples of supportive phrases to provide.	Includes real photos of the doctor’s office, medical instruments, and receiving a vaccination. Appropriate holding during the procedure can also be seen, which may be helpful for parents to view. Written by a certified Child Life Specialist and mother.
*JJ’s Shot to Save The World*	[62]	23.75/NA	✔ ^1,6,8,9^	--	✔ Vague explanation on how vaccines work. However, includes clear lists of evidence-based tips for parents and describes distraction. Includes some direct examples for child education (e.g., “explain that vaccines can pinch or sting, but that it won’t hurt for long and after they’ll be an infection fighter”). Highlights 4 tips to help children on vaccine day (be truthful, be calm, distract them, make it an adventure).	Written by Dr. Sanders and his wife, who have a daughter with needle fear. Features very child-friendly visuals and a number of helpful strategies including caregiver support during the needle appointment.
*JoJo Wonders, What Are Vaccines*	[63]	12.03/4.00	✔ ^1,8,9,10^	✔ ^c,d^	--	Features multiple pages dedicated to explaining how vaccines work. The book concludes with some helpful keywords for children.
*Judah Mackabee Goes to the Doctor*	[64]	32.98/NA	✔ ^1,6,9,10^	--	✔ Has what appears to be a “guided discussion” section to be read by the parent to the child. Includes a clear explanation on how vaccinations work with examples. Parent content is somewhat vague, and no explicit information is provided on how to manage needle pain/fear. The sole strategy is highlighted by describing what a character does after learning about how the needle can help protect his sister: “he takes a deep breath and holds out his arm”.	Framed within a religious lens, referencing values and lessons. Emphasizes also the importance of getting vaccinated to protect others who cannot (e.g., younger sibling). Includes child-friendly visuals.
*Kelly Gets a Vaccine*	[65]	12.48/NA	✔ ^1,6,9,10^	✔ ^b^	--	Very information-heavy (includes U.S.-based information on the cost of vaccines, why health care professionals get them first, etc.). Includes also some evidence-based strategies.
*Kelsey Hates The Needle*	[66]	22.41/9.99	✔ ^9^	--	--	Compares a vaccination to a magic wand carried by a fairy that can help wilted flowers get the water and nutrients they need to grow tall and strong, which is not an accurate concept (i.e., vaccinations do not help you grow tall or strong). Very few evidence-based strategies represented.
*Lions Aren’t Scared of Shots*	[67]	11.78/NA	✔ ^1,6,7,8^	--	✔ Includes a “Note to Parents” section with excellent, clear, and detailed guidance (e.g., how to prepare, walking through procedures, etc.). Calming, normalizing details are provided framed in the broader context (e.g., acknowledges that children can be fearful of many things related to going to the doctor, including needles). References helpful suggestions not covered in CPGs (e.g., choices, empowerment, good match with clinicians, preparation, deep breathing, modeling).	The book author is an MD in pediatrics, Clinical Professor in pediatrics, and has 2 children. The adult-directed info is written by a psychologist (PsyD) who works with children and parents in private practice and is “an author on many books and articles addressing the concerns of children and their parents”. A key strategy explored in this book is using guided imagery/imagination to cope with aspects of the doctor’s appointment.
*Little Shots for Little Tots*	[68]	17.01/6.24	✔ ^1,7,8,9^	--	--	Very little text, which may be appropriate for younger children. Includes child-friendly and colorful visuals.
*Maya Visits Her Doctor Vaccination*	[69]	12.99/2.99	✔ ^7,9^	--	--	Features the needle as an animated character within the narrative. Many procedures at the doctor’s office depicted in a child-friendly manner. The book ends with multiple-choice questions parents can ask their children about the story.
*Miss Violet Vaccine*	[70]	15.72/3.92	✔ ^8,9,10^	--	--	Features a large, animated needle character that wears a cape and provides information about getting a vaccination. Includes a few evidence-based strategies.
*Needle Day*	[71]	NA/0.99	✔ ^4^	--	--	Features stick-figure-type characters with little to no background setting depicted. A short e-book depicting very few evidence-based strategies.
*Nicky Goes to the Doctor*	[72]	5.93/NA	✔ ^1,9^	✔ ^d^	--	Includes a vaccination as one of several other parts of a doctor’s visit (e.g., eye test, reflex test, checking height, etc.). A disproportionally large needle depicted that may be interpreted as scary for children.
*Please Explain Vaccines to Me Because I Hate Shots*	[73]	24.29/6.13	✔ ^1,3,5,6,7,8,9,10^	✔ ^d^	✔ Includes an over 30-page Parent/Caregiver Guide with an extremely detailed and potentially confusing definition of high needle fear for a non-scientific audience. Presents suggestions for child education (e.g., bibliotherapy, preparation, taking child through list of strategies). Examples of distraction are provided, some of which are not evidence-based. “Vicarious extinction” is referenced and explained as modelling by an unafraid, coping individual. Exposure is also mentioned, but is construed as being more about having a neutral experience at the office/site. Included other problematic strategies/information (e.g., “play doctor” with exaggerated pain responses and ice being effective analgesia).	While several helpful and evidence-based strategies are represented, a few very scary visuals diminish their value. The parent guide is written by a board-certified child psychologist (PhD), credentialed play therapist, a mother, and a grandmother with 4 decades of experience. Includes a highly detailed and potentially confusing parent guide with inconsistent information (e.g., suggests being honest, but outlines two strategies that are not (e.g., false suggestion regarding “pores”).
*Pout Pout Fish Goes to the Doctor*	[74]	8.17/6.99	✔ ^8,9,10^	✔ ^d^		A few helpful and evidence-based strategies depicted. However, some scary visual depictions may promote fear in children.
*Puppy Needs A Vaccine*	[75]	NA/3.61	✔ ^8,8,9,10^	✔ ^b^	--	Several evidence-based and helpful strategies represented. Focuses on learning about vaccination, not veterinary care.
*Sammy the Shot*	[76]	10.48/NA	✔ ^1,6,7,8,9^	--	✔ Includes “A Message for Grown-Ups” section. Vague, but accurate explanation of needle fear. Connections made to parents’ tendency to avoid telling children they are receiving a needle. Parents encouraged to be honest and use the story to prepare the child before the needle. Other preparation strategies included rehearsal and role playing both before and after the procedure	Includes many helpful strategies such as education, as well as a helpful depiction of a character receiving the needle sitting in the caregiver’s lap. Includes an animated needle character, but child-friendly visuals overall. The author is an MD (pediatrician) who runs his/her own pediatric practice; described in “dear reader” section at the beginning. Describes own approach to shots leading to “calmer and less fearful reaction”.
*Sofia and the Shot*	[77]	16.06/6.81	✔ ^1,6,8,9,10^	✔ ^d^	--	Character goes on an adventure in her dream where she learns about vaccinations and their importance. Includes one very large image of a needle going into skin that may potentially be scary for children. Other visuals in the book are child friendly overall.
*Story of Vaccines*	[78]	15.65/7.1	✔ ^9^	--	--	Includes a somewhat lengthy and detailed explanation of the science behind how vaccines work. Includes a history on vaccinations and references the cowpox vaccine. Very few helpful strategies depicted.
*Thank You for Getting Your Shots*	[79]	17.16/6.58	✔ ^9^	✔ ^d^	--	Includes simple graphics with not much detail. Explains community immunity concisely. Features some large needle images that may be scary for children.
*The Berenstain Bears go to the Doctor*	[80]	7.91/7.99	✔ ^3,6,7,8,9^	✔ ^b,d^	--	One of the oldest books within the sample. Features many procedures at the doctor’s office. Depicts also a character’s arm appearing bent after receiving a needle, which may be scary to children.
*The Boy Who Overcame His Fear Of Needles*	[81]	15.31/NA	✔ ^7,8,9^	✔ ^b,c,d^	--	Some helpful strategies featured. Cover of book depicts a giant needle (larger than character), which may be scary for children. Reward (lollipop) is given before delivering the needle.
*The Magic Power-Ethan, Mia, and the Amazing Vaccine*	[82]	13.7/6.68	✔ ^1,6,8,9,10^	--	--	Includes child-friendly, colorful graphics. Many evidence-based strategies represented with a clear explanation on why needles are important. Features also siblings supporting each other.
*The Measly Virus*	[83]	10.46/2.6	✔ ^1,9^	--	✔ The entire adult section is devoted to explaining community immunity. Explains vaccines describing germs akin to rain and vaccines like umbrellas.	Describes an animated virus character as a “menace” that may seriously harm younger sibling or grandparent. Emphasizes the importance of getting vaccinated for herd immunity.
*The Pinch Pixie-Conquering Vaccine Fear*	[84]	15.24/3.8	✔ ^1,8,9^	--	--	Describes needle pain as a “pinch”, but acknowledges variability in children’s responses to the pinch. Emphasizes that protecting others by getting vaccinated is heroic. Child-friendly pictures are included.
*The Real Life Super Power: A Creative Story for Children to Help Boost Their Bravery about Getting Shots at the Doctor’s Office*	[85]	12.66/5.1	✔ ^1,8,9,10^	--	--	Features visuals that are not scary, but are somewhat disorganized and confusing (e.g., dentist clinic, doctor’s office, inpatient setting). Emphasizes the importance of getting shots to prevent getting sick and comparing it to a superpower. Multiple evidence-based strategies featured.
*The Scared Sheep Needed A Shot*	[86]	10.7/4.0	✔ ^7,9,10^	✔ ^b,e^	--	Needle pain initially described as “Stinging like a bee”; however, later, the character says that “it barely hurt”. Some helpful strategies are included.
*The Shots Book: A Little Brother’s Superhero Tale*	[87]	19.63/NA	✔ ^9^	--	--	Emphasizes the importance of getting vaccinated to protect others who are unable to. Few evidence-based strategies included.
*Vicky Gets a Vaccine*	[88]	25.77/9.99	✔ ^1,7,8,10^	✔ ^b,d^	--	Multiple evidence-based strategies are included; however, there are some visuals that may be scary for children (e.g., a dark and scary hallway with a vaccine sign).
*When a Shot Means a Lot*	[89]	22.99/9.99	✔ ^9,10^	✔ ^d^	--	A COVID-19-specific book that acknowledges changes in children’s lives during the pandemic. Explains that receiving a vaccine means everyone will be able to see each other again safely. A few pictures of large needles are included that may be a bit scary for children.
*Who Needs a Check Up*	[90]	6.92/4.79	✔ ^8,10,11^	--	--	Includes modelling and exposure with the character having a pretend appointment with a friend where they receive a pinch to mimic a needle insertion. The character is then able to go to the appointment. Includes a few evidence-based strategies.
*You are So Brave: Ellie and Leo Go to the Doctor*	[91]	16.06/NA	✔ ^1,4,5,6,7,8,9,11^	--	--	Includes many evidence-based strategies described or depicted clearly. Visuals are child-friendly and detailed. Ends with discussion questions about the story.

* NA. refers to a book format that was not available. ^1^ Upright positioning. ^2^ Numbing cream/gels. ^3^ Distraction: verbal. ^4^ Distraction: breathing. ^5^ Neutral wording. ^6^ Caregiver presence during needle. ^7^ Education: procedural information. ^8^ Education: sensory information. ^9^ Education: importance of procedure. ^10^ Education: in advance of procedure. ^11^ Exposure/modelling. ^a^ Restraint of needle recipient. ^b^ Simplified reassurance/false suggestion. ^c^ Scary written descriptions. ^d^ Scary visual depictions. ^e^ Apology to needle recipient.

## Data Availability

Data are contained within the article and Appendix A, Appendix B and Appendix C.

## Appendix A

**Table A1:** Screening criteria for identifying children’s picture book’s depicting needle procedures.

#	Screening Criteria		
1	English	Yes	
		No	
2	For ages 5 to 8 (inclusive)?	Yes, explain why: (1 needed)	Listed age range includes ages 5 to 8 Listed Reading Level: Kindergarten Text Included Other: ___________
		No, explain why: (more than yes needed, if = then yes)	Listed age range < 5 Listed age range > 8 Only illustrations/ No text Reading Level: preschool Reading Level: Grade 4+ Other: ______________
3	From the title, description, and/or preview, does the book appear to have content related to needles or a needle procedure (i.e., vaccinations, blood draws)?	Yes, explain why: (1 needed)	Needle related title Needle related description/comments Needle depicted in preview Other: _____________
		Yes, but content related to:	Diabetes (e.g., finger pricks, insulin injections) Cancer (e.g., biopsy, bone marrow aspiration) Other chronic illnesses Dentistry (e.g., needles for numbing) Veterinary Services (i.e., needle for an animal)
		No, explain why:	Sewing/Knitting needles Taking chances Other: ________________
4	Picture Book?	Yes, explain why: (1 needed)	Target age: 2 to 8 <1000 words More Illustrations than text ≤32 pages Dimensions ≤ 25 cm Other: ____________
		No, explain why: (more than yes needed, if = then yes)	More text than illustrations Has chapters >1000 words >32 pages Graphic Novel Other: _______________
5	Eligible to order?	Yes	
		No	

## Appendix B

**Table A2:** Coding scheme for children’s picture books informed by CPGs and systematic reviews on managing needle fear and pain.

Type of Book:	Information based/educational (No characters or narrative, just explanations or facts)	1
	Character/story based (consists of a narrative. e.g., character getting a needle procedure, character being educated about needles)	2
	Both information and story based (includes separate educational section and separate narrative-based story)	3
	Other (specify)	4
2. Presence of Needle procedure–is it explicitly described, explained, or pictured/shown:	ONLY described or explained (i.e., no needle or needle procedure is visually depicted)	1
	ONLY pictured/depicted (i.e., a needle/injection/similar shaped object is visually seen)	2
	BOTH described/explained AND pictured/depicted	3
	Other (specify)	4
3. Location of the needle procedure:	Doctor’s clinic/Physician’s office/Hospital/General Exam Room	0/1
	Home	0/1
	School	0/1
	Other (specify)	0/1
4. Needle procedure by itself or part of a set of procedures:	Only needle procedure shown/described/depicted	1
	Presence of ANY other procedures (e.g., throat check up, reflex test, stethoscope, etc. depicted explicitly)	2
	Other (specify)	3
5. Type of Procedure:	Blood draw/venipuncture (do NOT code unless explicitly mentioned, blood seen going in/out of needle)	0/1
	Vaccination/ immunization/shot (do NOT code unless explicitly mentioned)	0/1
	Unknown Needle Procedure (a needle is depicted/mentioned but vaccination/blood draw are not explicitly mentioned, and the pictures do NOT depict blood going in/out of the needle)	0/1
	Other (specify here if it does not fall into any of the above categories)	0/1
6. Others present during the needle procedure:	Parent (Mom)	0/1
	Parent (Dad)	0/1
	Parents (more than one)- specify	0/1
	Grandparent (one)	0/1
	Grandparents (more than one)	0/1
	Friend/peer (one)	0/1
	Friends/peers (more than one)	0/1
	Sibling (one)	0/1
	Siblings (more than one)	0/1
	Teacher	0/1
	Nurse (only code if nurse is explicitly mentioned through wording of story or depicted in a picture. A character looking like a nurse (e.g., wearing nurse hat and uniform) is NOT sufficient to be coded into this category)	0/1
	Pharmacist	0/1
	Doctor/Physician (only code if doctor/doctor’s office is explicitly mentioned through wording of story or depicted in a picture (e.g., character has name tag with “Dr. Bear”)	0/1
	Child life specialist	0/1
	Mental health professional (e.g., psychologist, therapist, etc.)	0/1
	Other (specify; includes other health care providers that cannot be coded into any of the above categories)	0/1
	N/A (No others present)	0/1
7. OTHER Strategies Depicted	Restraint of character receiving needle (e.g., being held down to exam table, being held too tight by another character)	0/1
	Application of ice (or other cold object (e.g., frozen bag of vegetables)	0/1
	Vapocoolants (i.e., cold spray)	0/1
	Oral Pain Medication (e.g., Tylenol, Advil, etc.)	0/1
	Tangible Rewards before or after the needle procedure. Can be mentioned, depicted, or both. Does NOT include verbal praise. (e.g., lollipop/sticker after needle, parent referencing going out to ice cream or buy a toy after the procedure)	0/1
	N/A (No Not Evidence-Based Strategies Depicted)	0/1
8. Evidence-Based Strategies Depicted	Positioning–Sitting upright	0/1
	Muscle Tension (e.g., description or depiction of tensing/squeezing muscles)	0/1
	Numbing creams, gels, and/or patches	0/1
	Distraction during the procedure–verbal (e.g., asking character an unrelated question during procedure, “what should we do after we’re done here?”)	0/1
	Distraction during the procedure–breathing toy (e.g., bubbles, pinwheel, etc.)	0/1
	Distraction during the procedure–media (e.g., showing a video and/or playing music)	0/1
	Distraction during the procedure–Other (please describe) (e.g., guided imagery)	0/1
	Neutral wording to signal the procedure (e.g., “here we go”, “here it comes”, “3, 2, 1…”)	0/1
	Presence of parent/caregiver character during needle procedure	0/1
	Character and/or reader education–procedural information (e.g., “now I am going to clean your arm”, “this part will take 2 min” etc.)	0/1
	Character and/or reader education–sensory information (e.g., “this may feel cold”, “you might feel a pinch”)	0/1
	Character and/or reader education–importance of needles/ purpose of procedure	0/1
	Character and/or reader education–in advance of procedure (e.g., at home, in the car, in waiting room, etc.)	0/1
	Exposure or “facing fears” in advance of procedure (e.g., showing character a needle before procedure, watching a video of needle before procedure, giving a toy a needle)	0/1
	Other (specify)	0/1
	N/A (None of the evidence-based strategies represented)	0/1
9. Distress behaviors represented (shown visually or described in text) by the needle recipient specifically:	Crying/Screaming (e.g., “Mr. Bear sobbed during the poke”, or depiction of tears, yelling, or screaming)	0/1
	Verbal Resistance (e.g., “I don’t want it”, “Stop!”)	0/1
	Requesting emotional or physical support (e.g., “can you hold my hand?”, “hold me!”)	0/1
	Information seeking (e.g., “Why do I need it?”, “will it hurt?”, “when will it end?”)	0/1
	Verbal expression of fear (e.g., “that looks scary”, “I’m afraid of needles”)	0/1
	Verbal expression of pain (e.g., “oww!”, “that hurts!”, “it stings”)	0/1
	Other (specify)	0/1
	N/A (no distress behaviors represented)	0/1
10. Distress Promoting Behavior directed towards needle recipient _____________________ If applicable, specify from: needle provider/ Parent/peer/sibling:	Restraint of character receiving needle (e.g., being held down to exam table, being held too tight by another character; this does NOT include being held in a gentle hug)	0/1
	Simplified reassurance and/or false suggestion (e.g., “don’t worry”, “it’s/you’re okay”, “it won’t hurt”, “it will be fine”) even if followed by helpful/evidence-based information	0/1
	Exaggerated or scary written descriptions of the needle and/or procedure (e.g., “the giant needle poked her arm”)	0/1
	Ominous or scary visual depictions (e.g., unrealistically large needle). If 1 in this category, specify rating of the book on a 1–10 NRS.	0/1
	Ominous or scary visual depictions RATING the book as a whole. 1–3: mildly scary or ominous 4–6: moderately scary or ominous 7–10: highly scary or ominous Code based on how ominous or scary a child would find the book as a whole. Do NOT rate each scary picture in the book. Rate based on the scariness of pictures in the book, NOT on the quantity of scary pictures. (i.e., a book with only one highly scary picture would get a rating higher than a book with multiple mildly scary books).	1–10
	Criticism (i.e., negatively evaluating character receiving the needle; e.g., “brave kids don’t cry”, “Why are you crying so much?”, “You didn’t use your breathing like I told you to”)	0/1
	Apology (i.e., verbalization of sorrow; e.g., “I’m sorry we have to do this”, “I wish I didn’t have to hurt you”, “we don’t like doing this either”)	0/1
	Other (specify)	0/1
	N/A (No distress promoting behavior)	1
11. Is there a “Note to Parents” or supplementary adult message provided related to needle procedures at the start, middle, or end of the book?	No	0
	Yes (describe briefly)	1

## Appendix C

**Table A3:** Coding scheme for supplementary adult material.

#	Category	Notes		If Yes:	
1	Definition/Explanation of Needle Fear	A definition, description, or explanation of the fear of needle procedures. Can include references to anxiety or phobia related to needle procedures. May also include references to the prevalence of needle fear.	0—No 1—Yes	If yes: 0—Seems inaccurate 1—Seems accurate (Please Describe) ________	
2	Vaccination Info–How vaccines work	A description or explanation of how vaccines work (e.g., what is in vaccines, how they build immunity, they keep you from getting sick, etc.). Does not include how they work on a community level (i.e., community/herd immunity or limiting spread of disease).	0—No 1—Yes	If yes: 0—Seems inaccurate 1—Seems accurate (Please Describe) ___________	
3	Vaccination Info–Community/Herd Immunity	A description or explanation of community/herd immunity (i.e., by getting vaccinated, you are protecting others). This includes mention of how vaccines prevent the spread of infectious diseases in communities.	0—No 1—Yes	If yes: 0—Seems inaccurate 1—Seems accurate (Please Describe) ___________	
4	Vaccination Info-Other	Other information specific to vaccinations that refers to their importance or function (e.g., history of vaccines, how many vaccines are required, etc.)	0—No 1—Yes	If yes: 0—Seems inaccurate 1—Seems accurate (Please Describe) ___________	
5	Visualization/Guided Imagery	Refers to using mental visualization or imagination as a helpful strategy for needle fear (e.g., picture leaves floating down a stream in a forest)	0—No 1—Yes		
6	Rewards	Refers to providing children with a desirable item or experience following successful completion of a needle procedure (e.g., ice cream, new toy, sticker, etc.)	0—No 1—Yes		
7	Expertise	Writer or writers of the book have expertise in guidance. This could include an advanced graduate degree (MD, PhD, RN, MSW, PharmD, etc.), clinical experience (e.g., work with children with needle fear), or lived experience (e.g., parent of child with needle fear).	0—No Expertise described 1—No relevant expertise 2—Yes, relevant expertise	If yes, please describe expertise (e.g., pediatric nurse, mom of child with NF, etc.)	
	Clinical Practice Guidelines				
	Category	Code any Accurate Mention of:	Reference	Y/N	If Yes, Describe
8	Avoid aspiration (for vaccinations only)	Aspiration should not be used during intramuscular vaccine injections as the technique can increase pain.	[13]	0—No 1—Yes	
9	Most painful needle last	As some vaccines are more painful than others, and many children receive multiple injections at a single visit, the most painful needle should be administered last.	[13]	0/1	
10	Positioning-Sitting upright	It is recommended that during the needle, children ages three and older sit upright rather than be lying in a supine position. This positioning has been shown to decrease fear and observed distress in children.	[13,14]	0/1	
11	Avoid restraining the child.	Forcibly restraining a child during the needle is advised against as it may increase the child’s fear.	[13]	0/1	
12	Muscle Tension	Muscle tension is recommended for children ages seven and above with a history of fainting. This occurs immediately before and during the needle	[13,16]	0/1	
13	Combination of Cold & Vibration (Buzzy)	The Buzzy Device, which applies vibration and cold above the needle insertion site immediately before and during the needle has been shown to be a promising intervention for reducing procedural pain and anxiety.	[18]	0/1	
14	Using topical anesthetics	Topical anesthetics block the transmission of pain signals from the skin (McLure & Rubin, 2005) and are recommended for children ages twelve and below. They can be in the form of numbing creams, gels, or patches.	[13]	0/1	
15	Clinician Education	It is recommended that clinicians administering the needle be educated on implementing needle pain and fear management strategies before the day of the needle	[13]	0/1	If yes, Describe: ________
16	Parent Education	Educating parents on how to mitigate their child’s pain and fear both before and on the day of needle is recommended before and on the day of the needle to increase use of interventions during the needle	[13]	0/1	If yes, Describe: ________
17	Child Education	Children ages 3 and up should be provided procedural (e.g., what will happen?) and sensory (e.g., how will it feel?) information and be taught how to use coping strategies. Such information should mostly be provided in advance. On the day of the procedure, children should be provided with neutral information and coping strategies.	[13]	0/1	If yes, Describe: ________
18	Parent Presence	Parents of children aged 10 and younger should be present during the child’s needle.	[13]	0/1	
19	Neutral Wording to Signal Procedure	It is recommended to use neutral wording to signal the impending procedure to children (e.g., “here we go!”).	[13]	0/1	
20	Avoid Reassurance & False Suggestion	Repeated and simplified reassurance and false suggestions (e.g., “don’t worry”, “it’s okay”, “it won’t hurt”) can increase children’s distress and pain.	[13]	0/1	
21	Distraction	It is recommended that distraction be used to draw children’s attention away from the needle. For children ages 3 to 12, verbal, video, and music distractions are recommended. Does NOT include breathing interventions (See Code #22)	[13,17]	0/1	If yes, Describe: ________
22	Breathing with Distraction	Breathing interventions, such as coughing, are recommended against. Instead, it is suggested that children breathe with a toy distraction during the needle (e.g., pinwheel, bubbles, etc.).	[13,17]	0/1	If yes, Describe: _______
23	Exposure-based therapy (EBT)	For children ages 7 and above with high levels of needle fear, EBT is recommended. In fact, EBT is considered the gold standard treatment for specific phobias such as blood-injury injection phobia.	[13,15]	0/1	If yes, Describe: _______
24	Applied Tension	For children ages 7 and above with high levels of needle fear and a history of fainting, applied tension is recommended rather than exposure-based therapy on its own.	[13,15]	0/1	
